# Attitudes and experiences regarding preventive strategies for the deaf population in Western New York

**DOI:** 10.1371/journal.pgph.0001056

**Published:** 2023-11-22

**Authors:** Lorne Farovitch, Carol Padden, Edwin vanWijngaarden, Benjamin Miller, Brian Leydet, Timothy Dye

**Affiliations:** 1 Translational Biomedical Science, University of Rochester Medical Center, Rochester, New York, United States of America; 2 Division of Social Sciences, University of California, San Diego, San Diego, California, United States of America; 3 Department of Public Health Sciences, University of Rochester Medical Center, Rochester, New York, United States of America; 4 Department of Dermatology, University of Rochester Medical Center, Rochester, New York, United States of America; 5 State University of New York College of Environmental Science and Forestry, State University of New York Upstate Medicine University, Syracuse, New York, United States of America; 6 Department of Obstetrics and Gynecology, University of Rochester Medical Center, Rochester, New York, United States of America; University of Canberra, AUSTRALIA

## Abstract

People for whom English is a second language, such as the deaf population, often have unequal access to health information and low health literacy. In the context of a wider study on risk of tick-borne illness in deaf communities, we explored barriers, opportunities, and nuances to accessible health information and communication among deaf people. Semi-structured qualitative individual and group interviews were conducted with 40 deaf people in upstate New York, to explore factors associated with health literacy and health information accessibility. Interviews were conducted in American Sign Language (ASL) by a deaf researcher fluent in ASL. Data analysis included the translation of ASL signs into English words, systematic coding, and generation of themes. A total of 21 interview events (mean time per interview = 41 minutes) were conducted. Two main themes and multiple sub-themes emerged from the data: 1) Layers of obstacles faced by deaf people confirms (or reinforces) exclusion; and 2) preventive information is unavailable or inaccessible to deaf people. Sub- themes identified in the results were perceptions of the deaf community and deaf culture, complex layers of obstacles faced by deaf individuals, the digital divide, the culture of communication, awareness of tick and tick-borne disease (TBD) diseases, importance of using certified deaf interpreters (CDI), health information dissemination strategies and collaborations with the education system, and physical/virtual community engagement. The data suggested several challenges to health literacy in the deaf population, including healthcare and education inequalities and negative perceptions of deaf people by both deaf and hearing people. Improving health literacy in the deaf population requires more interpreters who themselves are deaf (“certified deaf interpreters”), provision of health information in ASL, and a greater engagement with the deaf population by education and healthcare systems.

## Introduction

According to the World Health Organization, 360 million people worldwide have hearing loss [1]. Efforts toward reducing disease risk require a clear mechanism by which scientific findings can be communicated to diverse populations, including deaf populations who use a signed language as their first language. The majority society is audiocentric: vital health and other information is usually disseminated through auditory channels such as radio, un-subtitled television, and public discussion. This information is inaccessible for deaf people and further exacerbates the deaf population’s low health literacy [2], increasing risks for disease [3]. Furthermore, clinical communication systems do not typically provide accessible health information material in the primary language of the deaf population, which additionally disadvantages deaf individuals [4]. As a result, the National Center for Deaf Health Research recommends six public health actions to improve health equality for the deaf population: collaboration of public health entities with deaf sign language users, inclusion of deaf people in surveillance and health research, implementation of studies of the deaf population, encouragement of deaf individuals to participate in public health campaigns, encouragement of deaf sign language users to pursue careers in public health, and expansion of funding to support communication access costs for public health programs and research [5].

Owing to language barriers, deaf populations are frequently unaware of the risks and preventive strategies regarding common public health problems, for example vector-borne diseases. This unfamiliarity leaves them at risk of health disparities owing to their lack of information access. Although efforts have been made to reach other minority groups, there are no data on attitudes toward and practices regarding vector-borne diseases in deaf populations (a minority with invisible disability) around the world. The deaf population is a unique group of people who require different approaches in efforts to improve health disparities, primarily through language, but also in the consequences of late language acquisition [6]. In a study on health literacy among the deaf population in the United States, McKee et al. (2015) found that half the deaf participants had inadequate health literacy and that deaf participants were 6.9 times more likely than hearing participants to have inadequate health literacy [4]. Individuals with low literacy skills, however, may be more responsive if health education content and delivery methods are adapted to rely less on the written word [7].

To develop effective preventive strategies, more studies are needed to identify health-seeking behaviors and relevant socioeconomic factors for vulnerable populations and to develop adaptive approaches [8]. Data from qualitative interviews could help better elucidate health-related issues, and to determine the best methods by which to disseminate information among populations without access to information in their primary language. In the context of a wider study on risk of tick-borne illness in deaf communities, we explored barriers, opportunities, and nuances to accessible health information and communication among deaf people. The results of this study will be disseminated to the appropriate audiences as an integral part of science communication.

## Methods

### Ethics statement

This study was approved by the University of Rochester’s Research Subjects Review Board (RSRB), which determined that the research met the requirements for federal and University criteria for exemption (RSRB Study ID: STUDY00004208). Informed consent was obtained for all participants. Waiver of documentation of consent was approved by the RSRB and consent was provided by participants through American Sign Language.

Semi-structured interviews were conducted with 40 self-identified deaf individuals from Western New York (Buffalo, NY, or Rochester, NY) to evaluate health information communications. The age of participants ranged from 25 to 73 years; most self-identified as female (n = 24; male n = 16) (Table 1). A total of 40 participants were interviewed from March 2020 to April 2020. A total of 21 interview instances were conducted in ASL, each lasting an average of 41 minutes.

**Table 1:** Study demographic information.

	Total (n = 40) n
**Age (years; median, range)**	33.5
**Sex**	
Female	24
Male	16
**Ethnic Background**	
Caucasian	32
Asian	4
Black	1
Latino	3
**Highest Education Level**	
GED	1
High School	2
Associate degree	5
Bachelor’s degree	17
Master’s degree	11
Doctorate degree	4
**Preferred Health Information Material**	
ASL	7
ASL and Written Language	7
ASL and Pictures	2
ASL, Written Language, and Pictures	6
Written Language and Pictures	9
Written Language	9

Participants were recruited using Facebook via a post on a deaf local organization’s page and word of mouth via snowball sampling (subjects who had participated in the study were asked to identify other potential participants and to pass on to them the researcher’s contact information). This approach was considered the best method of recruiting deaf individuals. A relationship with the deaf community was established prior to the start of the study: the lead researcher on this study engaged with the community as a community member and an educator for six years through multiple organizations, attending deaf events, and interacting with the deaf community to raise awareness about various health issues. The inclusion criteria were deafness, 18 years old or older, and residence in Rochester, NY, or Buffalo, NY. Participants were unpaid. American Sign Language (ASL) was used to collect demographic information from participants at the interviews.

Eligible participants contacted the researcher by email about study participation using recruitment materials posted on the researcher’s Facebook account via deaf pages/groups. The primary language used by the deaf population in the United States is ASL. The first author and principal investigator for this project is culturally deaf and fluent in ASL; therefore, an ASL interpreter was not needed for the interviews. Following Breslin’s method, ASL interpreters assisted in the process of translating ASL to written English to explicitly describe themes and participant experiences when interpreting the data codes [9]. An information sheet was sent to participants by email. Information about informed consent was given to participants in both written English and ASL. Based on previous research on populations for whom written consent is not appropriate, participants gave their verbal consent, which was recorded by the interviewer. Participants were informed about the research goal prior to the qualitative interviews through information sheets and recruitment flyers. Innovative materials were developed specifically for each interview location based on data collected using the rapid qualitative inquiry method [3]. The interviews focused on key questions about the deaf community, knowledge of tick-borne diseases (TBDs), health literacy status, and health information accessibility. The interviews were recorded using video chat with FaceTime, Zoom, or Sorenson/Convo. The researcher used an encrypted personal laptop to video record the interview, which was then uploaded into Dedoose (a cloud-based qualitative analysis system, a web application for managing, analyzing, and presenting qualitative and mixed method research data, Version 8.3). Dedoose was used to analyze the qualitative data and to generate codes to gain insights into the development of ideal materials, the strength of the communication network, and health literacy regarding vector-borne diseases in the deaf population.

We conducted five semi-structured group interviews including 24 participants and 16 additional individual interviews of approximately one hour each which used the same instruments and protocol. No participants refused to participate or dropped out during the study. The video interviews were reviewed to identify reoccurring concepts, ideas, and themes. A codebook structure was developed based on four components: the code, a brief definition, a full definition, and examples [10]. Etic and emic codes were identified and labeled as such. An iterative code tree was developed and used to analyze data thematically [11]. Most codes were words rather than phrases or paragraphs to facilitate the process of coding English words onto ASL signs without losing any meaning during the translation. The interviewer coded all codes directly onto the videos in Dedoose rather than in the transcripts to prevent any loss of information in translation from ASL to English. The codebook was developed from the recurrent themes that emerged from the interviews. The themes were developed from the topics discussed most frequently in the interviews. The coding system was developed by identifying core themes and subthemes. The themes were reviewed by analyzing the patterns of codes and translating codes into English from ASL. The study results will be disseminated to the appropriate audiences as an integral part of science communication.

## Results

Two main themes and multiple sub-themes emerged from the data: 1) Layers of obstacles faced by deaf people confirms (or reinforces) exclusion; and 2) preventive information is unavailable or inaccessible to deaf people. Sub- themes identified in the results were perceptions of the deaf community and deaf culture, complex layers of obstacles faced by deaf individuals, the digital divide, the culture of communication, awareness of tick and tick-borne disease (TBD) diseases, importance of using certified deaf interpreters (CDI), health information dissemination strategies and collaborations with the education system, and physical/virtual community engagement (S1 Table).

Within Theme 1 (layers of obstacles and exclusion), the most common prevailing problem that participants noted is the systematic oppression of and discrimination against the deaf population. The theme of negative perceptions of the deaf community was repeatedly mentioned by participants to express concerns about a lack of understanding of the deaf population. Participants shared their experiences of encountering hearing people with either negative or positive attitudes toward deaf individuals. Many participants expressed frustration about the negative perceptions of people outside the community:

*Interviewer: “What is the attitude of hearing people toward the deaf community?”*

*Interviewee #5: “Stupid.”*

*Interviewee #3: “Focusing on the ear.”*

*Interviewee #2: “Deaf people can’t.”*

*Interviewer: “Why do hearing people have that attitude?”*

*Interviewee #5: “Doctors focus on the ear and the money; they can make money off trying to cure the deaf people. They do not look into the culture, the language, and the value—they just look at the ear. The majority are hearing, so they feel they need to fix what they see is broken because they are not the same as the majority, but they don’t realize all of our other sense work just fine.”*

*Interviewee #2: “It’s just about the ear and that is the only thing that doesn’t function; all of our other senses function just fine. We can do anything else, just not hear.”*

*Interviewee #1: “I think maybe it is not necessarily fear but definitely a shock and naivety about what to do, because they see not being able to hear as a barrier to communicating with a person. If you can’t communicate with them, you can’t develop a connection, so they feel at a loss about what to do. They can’t understand them; that’s the basis of human connection and if they can’t develop a connection, it creates a separation and that feeling of ‘You are different from us.’”*

*Interviewee #3: “I agree with her, and in general humans are creatures of habit—oh, it’s snowing—yes, so humans are creatures of habit. When someone different appears who is not within their ‘norm’, they don’t know how to react. I think it is that feeling of not being able to connect and not knowing how to react to something when it is not within our regular routine, habit, or whatever we are used to.”*

Participants stated that they felt they had not received enough support from both inside and outside of the community and had found much information inaccessible, for example:

*“I realize that our deaf community is diverse in their levels of understanding and their willingness to ask for clarification. Some will be passive and not ask because they do not want to be looked down on as less intelligent and have their pride hurt. They would rather nod and smile instead of fully understanding.”*

*“I have a question*: *do CDIs* [Certified Deaf Interpreters, who are trained ASL interpreters who are also deaf] *and hearing interpreters both get paid*? *I feel like it is a waste*. *I don’t mean to worry about the government and money*, *but I just see there are a lot of budget cuts for residential deaf schools*, *which is sad*, *but are they paying for two interpreters*? *The hearing interpreter should be able to adjust and make it work*.*”*

Almost half of participants brought up topics about CDIs (Certified Deaf Interpreters) and expressed the importance of CDIs due to culture and nuances of ASL communication.

*“It really breaks my heart to see. Sometimes I am so frustrated by interpreters. This one time, I was at an appointment for hip replacement surgery, and there was both a hearing interpreter and a CDI. I was floored watching how they worked, and I really learned that day the difference between the two. My daughter was there with me, and we were both watching the hearing interpreter. Now, I am great at catching and reading facial expressions and I noticed my daughter make a face, so the CDI interrupted and took over. The CDI took a word that the hearing interpreter had fingerspelled and explained to me what that word meant. I was impressed! It was like having an ASL dictionary there in the room with me! The CDI was able to clearly explain to me what the hearing interpreter had not.”*

*Interviewer: “Can you tell me more about how CDIs are clearer than hearing interpreters?”*

*Interviewee: “Hearing interpreters fingerspell so many words without actually explaining what they mean, whereas the CDI will explain the words. For example, the word ‘cancer’. The CDI asked me once if I knew what it meant. I said that I knew there were different kinds. The CDI then proceeded to explain to me what cancer was. When I looked back at the hearing interpreter, s/he just kind of nodded and verified that the CDI was right. When I asked the hearing interpreter why s/he did not do that, s/he said ‘Well, I fingerspelled the word, so I thought you knew what that meant.’ I am just the patient, though, here about a hip surgery. I am not the expert. There were several times throughout the appointment where the CDI kept having to jump in and explain things to me. It seemed like the CDI knew more than the hearing interpreter.”*

*Interviewee #5: “I think it is ASL with context, cultural information; they [CDIs] are from the same community, facial expressions, showing severity, expressing different extremes. It fits with the community.”*

*Interviewee #4: “CDIs also know more slang and can catch those things; hearing interpreters miss those things.”*

*Interviewee #3: “Spatial reference is also something CDIs use well; there was that one viral meme of the CDI who was interpreting for ‘social distancing’ and everyone understood the meaning clearly because of his use of space, even hearing people. His use of space made sense visually and we did not have to do any additional translation in our minds to understand the meaning. So yes, that context and culture is there.”*

As an example of access to health information around a particular disease category, participants were asked about their knowledge of ticks and TBD; the majority of participants had some awareness of ticks and TBD, whereas almost half of participants knew nothing about ticks or TBD. The two underlying causes of this lack of awareness were a lack of ASL information about ticks and TBD and the belief of some participants that ticks and TBD would not affect them. Most participants mentioned that they knew about ticks and TBD because they had dogs, relatives with TBD, or often hiked or camped. All participants emphasized the need to provide information in ASL along with pictures like infographics. Some participants said that the information should be displayed in ASL via videos in healthcare settings and through various platforms like official government websites. Additional information dissemination methods that participants mentioned in the interviews were flyers, QR codes, brochures, infographics, news, hotlines, physicians, mail, email, documentaries, churches, physicians, social media, word of mouth, and articles. Many participants indicated the importance of one-on-one sessions and workshops to spread and discuss health information. Many also mentioned the need to raise awareness about ticks. The participants have touched on a topic of the digital divide. The digital divide in this century is a concern because not everyone has access to technology especially in marginalized population:

*“I work for [name of company] video relay service and I notice that those deaf people who do not have access to resources—as she just mentioned—have less information and they struggle. I struggle to help them as well because they just do not have that fundamental knowledge related to technology, resources, skills, and vocabulary. Deaf people who have access to resources and technology tend to understand more; there is a big difference that I notice between these two groups every day in my work.”*

Many participants believed that health education about ticks and TBD and other important information should be incorporated into the education system.

*“Right, well—I mean kids in school should learn while they are in school, elementary school, etc. They should learn there; develop habits of getting information and then go from there.”*

*“Really, it’s all about the big picture and there are many variables. For example, there is no standard definition of deafness. There are many different levels of hearing loss as well as many identities. I personally work in a college admissions department. How do I determine if students qualify as deaf or hard of hearing? If someone went to a school for the deaf, does that mean they automatically qualify? But what if that person technically has less of a hearing loss than someone who was mainstreamed? That mainstreamed person might be viewed as ‘not deaf enough’ because they did not go to a deaf school or receive services. But how is that fair? The mainstreamed student might have lived in a rural area where there were no interpreters, and the local professionals knew nothing about deafness. Do I tell that student, ‘Too bad, you don’t qualify’? Other colleges could have different definitions or criteria for determining if a student qualifies. Our college follows Congress’ definitions of deaf and hard of hearing, but how did Congress come up with their definitions? I am sure that nobody that identifies as deaf or hard of hearing helped Congress develop these definitions. So, in the grand scheme of things, the education system needs to develop a standard because it currently does not have one. There is no way to really know if the education at schools for the deaf is the same as that at hearing schools, because hearing schools are highly varied. There is a vast difference between public schools and private schools. Some parents have the luxury to spend $100,000 a year on their child’s education while other parents only have the option of sending their child to public school and hoping for the best. Like I said, there are just so many factors involved in education. Now, to take a deaf child and all the extra factors that should be considered for their education and to throw them into a public-school setting, on top of the other factors I just mentioned, oh my goodness! How can we help with all of that? It seems literally impossible.”*

Some participants mentioned that if anyone wants to raise awareness about ticks and TBD, they need to enter the community and be engaged with both the real community and virtual community via social media like Facebook and Instagram.

*“I think continuing to work in the community is important. An actual job. Many depend on SSDI [social security disability insurance], so their opportunities to get out and be visible are limited. They go into Wegmans and shop, and they are in and out, they’re not interacting with people. If someone is working there, they’re not having an ongoing relationship and building relationships with other people that would help them learn more about you.”*

*“Go to events hosted by and for the deaf community… take an ASL class. Be an advocate. If you see someone spreading misinformation about deaf people, correct them. They are a few ways you can be welcomed into the deaf community as an active participant.”*

## Discussion

The deaf population is characterized by multiple subgroups rather than one organized nationwide system. For many years, the deaf population and sign language have attracted stigma, discrimination, and negative attitudes [12]. These negative attitudes and stereotypes toward the deaf community cause multiple layers of negative effects [13]. Resource inequality and negative perceptions toward the deaf population accumulate to have small but important negative effects on overall health literacy in the deaf population. Disparities in the education system are particularly important. The education system in the United States is heavily rooted in a monolingual society [14]. The education system must be addressed to prevent the domination of a monolingual and monocultural system. Participants mentioned that the education system has failed the deaf community, as resources are not tailored to various types of deaf individuals.

Many comments and ideas expressed by the study participants identified the true root of the lack of awareness of ticks, TBD, and other health information as systematic oppression, or audism. Another example of audism is a deprivation of access to information through qualified interpreters. The lack of a quality interpreter service is common. Sometimes, the information received through interpreters is not sufficiently clear and certified deaf interpreters (CDIs) are needed to mediate deaf culture and language. The access to information is liberating, and the denial of it is oppressive. Participants expressed frustration with the constant need to self-advocate and with other struggles. Participants note that it is exhausting to frequently have to speak up for oneself; therefore, deaf people prefer to stay within the deaf community and develop resistance to any messages from hearing people, including messages from hearing people in the healthcare and public health systems [15].

Visual learning information channels are readily available; unfortunately, deaf individuals, especially deaf children, are frequently denied the natural “path of least resistance” [15]. The pressure to adapt to the environment does not permit optimal development as a person [15]. The inhibition of deaf people’s natural learning mechanisms leads to language deprivation and poor quality of life. Language strategies for deaf people tend to focus on improving residual hearing using technologies like hearing aids and cochlear implants with hearing and speech training, and neglecting the natural potential of the visual learning method [15]. According to social cognitive theory, an auditory-dominant world has substantial effects on an individual’s lifestyle [15]. The term “audism” describes the concept of supremacy and hegemonic privilege claimed by audiocentric people [16]. Audism frequently occurs when audiocentric people within the system believe that deaf people cannot control their lives and promote schemas and structures that drive audiocentric privilege [16].

The definition of “grassroots” in the deaf community is “really deaf,” which means untouched by hearing ideologies [17]; thus, the form of information being exchanged is different to the approach by educated deaf individuals. Furthermore, the concept of the segregation between grassroots and educated deaf individuals within the deaf community is based on internalized oppression and internalized dominance caused by linguism, a language-based form of discrimination that is promoted by the monolingual society in the United States.

Of the respondents, 71% showed at least minimum knowledge about ticks or TBDs, which is approximately 24% lower than the study that tested for TBD knowledge in the hearing population [18]. Although data from qualitative research cannot be directly compared with data from quantitative research, the lack of research on this subject has limited the potential of understanding the severity of the situation. The lack of sufficient data shows the magnitude of this issue. Information about ticks and TBD is inaccessible to many deaf individuals because, as the participants pointed out, there are no ASL videos about ticks and TBD. The lack of access to information prevents deaf people from understanding the dangers of ticks and TBD. Information must be tailored to deaf people so that they can learn effectively.

Deaf individuals perform lower on reading comprehension than individuals who have English as their first language. Health literacy also describes an individual’s ability to understand and use healthcare information to make appropriate health decisions; for deaf people, this ability should be measured with their first language (ASL), and should take into account the failures of an education system that does not accommodate the needs of this population [4]. Most participants in this study explained that many deaf people could understand health information and make appropriate health decisions if they received health information materials in ASL.

Technology provides a unique way to reach out to different audiences within a community through social media. However, this strategy involves substantial work and an understanding of various backgrounds and cultures. The process of community engagement can expand the communication network within the real and virtual community. One participant shared an important tip to mitigate the digital divide within the deaf community by reaching out to deaf individuals through video relay services such as Sorenson, Convo, or Purple, because nearly every deaf person has a phone number affiliated with one of these services. Therefore, essential health information could be disseminated using this method. The participants also mentioned that information should be passed on to their community through their deaf leaders; thus, the process of community engagement to identify key stakeholders is essential.

The development of a structure and flow of communication and information within the community is the key to raising awareness about ticks and TBD, and would ensure that individuals who do not have access to technology or have low reading comprehension can access health information [19]. Participants stated that the deaf community is very large but intimate; therefore, the diffusion of information throughout the community means that many people are kept informed despite the digital divide. The digital divide is another form of oppression through technology that is based on unequal access to resources associated with technologies [19]. Participants expressed concerns about low socioeconomic status in the deaf population; therefore, the risk of a digital divide is high among this population. Overall populations in the nation with low socioeconomic status have been shown to be underserved in their access to the Internet and computer; therefore, their access to the majority of health information is hindered [20].

## Strengths and limitations

One of the strengths of this study is the strong relationship between the deaf community and the interviewer. The interviewer is culturally deaf and fluent in ASL, so was able to recognize all the important nuances in the conversations and include them in the data analysis process. Another strength is that the study met all four quality criteria for the trustworthiness of qualitative research: credibility, transferability, dependability, and confirmability [21]. The credibility of the research (authenticity, richness, idiographic statements, and honesty) was consistent owing to prolonged engagement (6 years of community engagement) and persistent observation of characteristics and elements in the study. Confirmability was established through an audit trail by providing a rationale and descriptions throughout the process of data collection, data analysis (codes and themes), and interpretation. The study transferability was strong owing to the thick description of behavior, experiences, and context in the various participant narratives. The details of the sample size, sample strategy, demographics, socioeconomic factors, inclusion criteria, interview procedure, themes, and excerpts were described thoroughly. Interviewer, participant, procedural, and sampling biases were avoided. Participant and interviewer biases were avoided by asking open-ended questions, and procedural bias was avoided by not rushing participants to provide responses. Sampling bias was limited because the researcher was engaged with and immersed in the deaf community and was able to reach out to participants with diverse backgrounds. The sample had a large age range (25 to 73 years) and a diverse educational background (General Educational Development exam [GED] to PhD degree). The ethnicity of participants was broad: Black, Asian, Caucasian, and Latino.

A study limitation was that only one researcher developed the codebook, which meant that inter-rater reliability could not be tested. Two of the four credibility criteria (triangulation and member checks) were not met.

## Implications for policy & practice

The identity of an individual is a quest to manage the bicultural existential tension of values in various groups, which is dominated by the system [22]. Therefore, community engagement and understanding deaf culture is essential to the process of understanding the deaf population’s capability to understand and adopt healthy behavior.

The findings from this study support the six public health recommendations to make public health more accessible and equal for the deaf population as identified by the National Center for Deaf Health Research [5]:

1) Public health entities must collaborate with deaf sign language users, preferably a certified deaf interpreter (CDI), to optimize and utilize the potential of health information materials by translating the materials into sign language.
2) Engage with the community to include diverse and marginalized populations in surveillance and health research and 3) include more deaf participants in public health.
4) More studies are needed on the deaf population, with assistance from CDIs, to inform the process of development of health information.
5) Build communication networks among the community and increase opportunities for deaf individuals to pursue careers in public health.
6) Advocate for funding to support communication access for public health programs and research.

## Conclusions

The assessment of health information materials has highlighted the importance of tailoring materials to diverse audiences, including the deaf population. Populations other than the deaf population may also benefit from approaches that present health information from different perspectives to improve understanding [23]. The process of communicating with communities by tailoring health materials could increase the effectiveness of preventive strategies for TBD or other diseases. Health information must be accessible to various populations within a country to make the healthcare system and other systems more inclusive and equal by providing information in ASL, infographics, subtitles, transcript, voice-overs, and one-on-one services such as hotlines. Socioeconomic disparity within the deaf population is another issue that leads to deprioritization of their concerns about ticks and TBD. Access to health information through various channels must be free because of the low socioeconomic status of the deaf population.

The data from these qualitative interviews indicate that the best ways of disseminating information among populations with no access to information in their primary language is to tailor health information to particular groups. This process could make health information more accessible, not just to the deaf population but to everyone. Future research on the deaf population and health information accessibility may lead to a better understanding of how to make healthcare systems more accessible to diverse audiences [24]; however, tailored health information accessibility is futile if the issue of systematic oppression is not addressed. The main problem that the deaf community faces has its roots in larger-scale, societal-level factors. A structural change in the monolingual health system would reduce the systematic oppression of marginalized populations. One effective change would be to ensure that the system is administered by individuals from diverse populations, such as Americans of color or disabled people [14, 25].

## Supporting information

S1 Table Deaf project codebook, with detailed descriptions of each code, frequency of code, and representative quote. (DOCX)
