# Supplementary material for: Attitudes and experiences regarding preventive strategies for the deaf population in Western New York
Source: PLOS Glob Public Health. 2023 Nov 22;3(11):e0001056. doi: 10.1371/journal.pgph.0001056 (PMC10664903; doi:10.1371/journal.pgph.0001056)
Supplement: S1 Table — (DOCX) [file pgph.0001056.s001.docx]

| **S1 Table: Deaf project codebook, with detailed descriptions of each code, frequency of code, and representative quote** | | | | |
| --- | --- | --- | --- | --- |
| **Parent code** | **Child code** | **Description** | **Code Frequency (% participants)** | **Representative Quote** |
| **Community** |  |  |  |  |
| Community Negative | Gossips/Backstabbing/Negativity | | 38% | well in the grassroots Deaf community (clarifies sign for grassroots) there's a lot of gossip. like people have nothing better to do... and i think that’s a big problem we are having right now. especially when it comes to stereotypes that hearing people make about the Deaf community. people gossip because they have nothing better to do to improve upon themselves |
|  | Mental Health | Mental health issues that are prevalent in the community. | 24% | I think it depends on where they live. If the Deaf person does not have good living circumstances, they tend to not have good health. There is also the mentality that it is the person's own doing and that this is the way they always have been and always will be. There is not a lot of support to help Deaf people overcome that mentality and to learn to live a healthy lifestyle. Mental health disorders, such as depression and anxiety, are prevalent in our community. There are various cliques within the community, and this can cause some individuals to feel excluded from certain groups. In turn, this causes some community members to feel upset or anxiety. I think if everyone came together as one large group, instead of having smaller exclusive groups, this would lead to better overall metal health for everyone. |
|  | Cliques | Groups that reject other groups because they do not share similar interests or privileges. Not enough deaf individuals that bridges between high education level with low education level. | 29% | [Name of city] is a very cliquey community. The grassroots people are typically left out, that's my perspective. I meet people and they've lived here all along, but I have never seen them before! Especially with the club, if you're involved with them then you are included. If you aren't then you get left out. There are people that I have never met before and I’ve been here since the 90's. It's crazy they've been here their whole lives and I meet them for the first time! |
|  | Bad eating habit |  | 19% | I think diet. What people eat. Yeah. How they eat. Quite a few - I've noticed low-income people - and this is true for both the Deaf and the overall community - what they buy to eat is really iffy: fast food, processed food, Kraft mac and cheese. Cheap food - minimal vegetables, minimal fruit. It's not great. I understand why they eat that way: it's easy to get, it's easy to eat, it's cheap. That's tough. I think that's the main health thing. I don't want to call it obesity although that's one result. |
|  | Don't make enough money |  | 24% | I think continuing to work in the community is important. An actual job. Many depend on SSDI, so their opportunities to get out and become visible are limited. They go into Wegmans and shop and they're in and out, they're not interacting with people. If someone's working there, they're not having an ongoing relationship and building relationships with other people that would help them learn more about you. |
| Community Positive | Large community |  | 62% | [Name of city] has a large Deaf community, and there are a lot of hearing people that have been exposed to Deaf people, so it feels comfortable. So, when I meet a new hearing person it's not so awkward, like they don't know how to interact with me, which always feels great. It's like sometimes I forget I am deaf here in [name of city]. |
|  | Many subgroups | Lot of experts in their field that able to support others. | 52% | There's a diverse range of students. I enjoy seeing a variety of backgrounds and cultures that everyone brings, there are different opinions and ideas based of their experiences and I may agree or disagree, but I still appreciate that. |
|  | Network | Lot of information available to share, lot of experts available to support each other | 33% | At any time if I ask for information about something, let's say gardening, the Deaf community will go out of their way to make sure I understand what needs to be done or how to do that specific thing. Another example is if I want to know about hiking trails, everyone is quick to throw out suggestions and say, "Hey this is a great place!" They really offer all of the information they have about whatever I am asking for, I always feel very supported. Let's say someone wants to know about job opportunities, people will go ahead and get you connected with whoever they may know. If someone knows someone at the university, they will connect them to that person and then they can have an opportunity because of the shared networks and connections. [Name of city] is really amazing in that way. |
|  | Communication | Lot of people are easily communicable and able to share essential information | 48% | That affinity or knowing each other or understanding what it's like to be deaf. you know, I moved here when I was 27. I'd been mainstreamed to that point. I was the only deaf person. I had a few Deaf friends but we only got together once in a while, we weren't together all the time and I didn't sign until I got to [name of university]. When I got here all of a sudden, I was involved in a larger Deaf community and wow it felt good. I felt emotional support, I felt very connected - I didn't have to explain myself. My frustration really went down, my enjoyment of life went up and wow - it was freeing, it was liberating in many ways. |
|  | Lot of resources |  | 29% | For me I do feel very fortunate to have the Deaf community here in the city. There are not many communities in the United States that have the resources and community that we have here in the city. The depth of knowledge that is here, the depth of Deaf culture, the ability to go out and interact with the hearing community when you’re going to the store and just out and about. It is better here than other places in the United States in general, there are a lot of positives about the community. |
| Deaf Culture |  |  | 24% | A lot of it is related to participating in a group. Being able to follow the information and not having things go over my head, not missing things. With deaf groups I can follow the information. With hearing groups I'm often missing a lot. It's tiring having to advocate for myself, asking people to repeat themselves, asking what's going on, relying on them to tell me important information. Maybe I don't know it's there but I'm relying on them to tell me. Deaf groups, the information is there. They know - it's a more instinctive sharing and understanding. There's no condescension or thinking I'm stupid as in why don't you know that there's no attitude like that. Hearing people - I wouldn't say it's attitude so much as I feel it's more effort for them. I feel not guilty, but I feel like it's more effort for them. They're working harder to include me. With the Deaf Community it's no added work, it's automatic. It's instinctual. |
| **Knowledge** |  |  |  |  |
| Physicians Visit |  | Using physicians' visits as baseline to understand their health status. | 43% | I have a couple of doctors I follow up with every 3 or 4 months, I have diabetes and high blood pressure. |
| Tick Knowledge | Aware about ticks and/or tick-borne diseases | The individual does know something about ticks and/or their diseases associated. | 71% | all I know is Lyme disease and ticks. yeah, i know that Lyme disease causes pain Interviewee #2: I know that you’re not supposed to rip the tick out of your skin with your hand because you want to make sure you get the whole thing out-- so that you don’t leave the head in which can cause Lyme disease [Interviewee #1 agrees] |
|  | Is not aware about ticks and/or tick-borne diseases | The individual does not know anything about ticks and/or their diseases associated. | 38% | Interviewee: My landlord told me. They told me not to go in that area because there are ticks there and to be careful. If my dogs go over there, I have to give them a bath. They just said that the ticks are dangerous, I didn't really know what that meant and why they were dangerous, but I believed my landlord. I don't know. Interviewer: Did you look into it and try to find out more information on your own? Interviewee: No. Interviewer: No? Why? Interviewee: I just got the information from my landlord and took it for what it was. I thought to myself hmm, ticks? Ticks are not that serious |
| Cause of Tick Awareness | Dogs | The individual has dog which is usually get bitten by ticks often. | 33% | it's not something that kills you immediately B: right people minimize it-- they aren’t really aware unless they have a dog or some other pet. if not, then they don't care much to learn more |
|  | Camping/hiking | The individual is interested in camping which is exposed to ticks more often. | 43% | when people are hiking in the woods they are exposed to the elements- including animals. The brother of an ex-boyfriend of mine got Lyme disease from hiking |
|  | Bitten by ticks |  | 29% | I was hiking at a national park and had a tick on my knee, luckily the head was not in my skin, but I was still paranoid and wondered if I should go to the doctor. But because the head didn't go into the skin it was ok. |
|  | (-) No ASL info related ticks) |  | 38% | No there's no ASL info for tick |
|  | (-) It doesn't have any effect on my life | | 33% | It just hasn't. It hasn't affected me, my family, a loved one, a friend, my entire life. But if ticks did finally have an impact on me, for example if someone did become infected or there was some significant potential impact to someone's health, I may pay more attention to it, but as of now it hasn't. And it's like, "If it ain't broke, don't fix it" kind of thing. So, I will just continue this way until something comes up. |
|  | Relatives with tick diseases | The individual has family member or friends with tick disease. | 24% | My niece got bit 3 or 4 times by ticks and at first she would just not tell anyone and keep it to herself. She would just pick it off herself, it would become inflamed and infected then she would have to go to the doctor. |
| **Accessibility** |  |  |  |  |
| No access to information |  |  | 43% | I work for [name of company] and I notice for those Deaf people who don't have access to resources as she just mentioned know less information and they struggle. I struggle to help them as well because they just don't have that fundamental knowledge related to technology, resources, skills and vocabulary. Deaf people who have access to resources and technology tend to understand more, there is a big difference that I notice between these two groups every day in my work. |
| Health Information Material | Internet |  | 76% | The internet, a google search. Interviewee #2: Me too, google. Interviewee #3: I go to google. |
|  | Social Media |  | 67% | Interviewee: There are never any workshops but there should be. Reading things with all of these words and terminology doesn't work. Interviewer: What about Facebook? Interviewee: Facebook is the same, that’s "hearing language." It needs to be put in a way Deaf people can understand it. Like how you do it. You will post a video signing the information, when I watch that I actually learn something! That is helpful, I can take in the information and understand it. |
|  | Word of mouth |  | 57% | So now I'm talking about [name of city]. Growing up, my parents belonged to a bowling league. I noticed that Deaf people love talking about health, medicine... Well, health and medicine, money, and, um, relationships. And I would just watch them talk. It could be good or bad, like maybe they're sharing wrong information, or they have correct information that could help someone. |
|  | Physicians |  | 52% | When Deaf people have to go to the hospital or their doctor that would be a great starting point to let them know that there is information available in ASL. It is possible they would look into it on their own after being told it is available and understand their current medical or health situation better. Then they can share their personal experience with others. |
|  | Workshop |  | 57% | The study I participated in about weight at [name of university] was helpful because it was free. I was able to have my blood drawn to check things related to my health like my cholesterol. It was an opportunity for me to find out about my health and if I am doing okay. Prior to that study, I was not very aware of my overall well-being. |
|  | ASL |  | 100% | Interviewee: I know. It really breaks my heart to see. Sometimes I'm so frustrated by interpreters. This one time, I was at an appointment...and there was both a hearing interpreter and a CDI. I was floored watching how they worked, and I really learned that day the difference between the two. My daughter was there with me, and we were both watching the hearing interpreter. Now, I'm great at catching and reading facial expressions and I noticed my daughter make a face, so the CDI interrupted and took over. The CDI took a word that the hearing interpreter had fingerspelled and explained to me what that word meant. I was impressed! It was like having an ASL dictionary there in the room with me! The CDI was able to clearly explain to me what the hearing interpreter didn't. Interviewer: Hearing interpreters fingerspell so many words without actually explaining what they mean, whereas the CDI will explain the words. For example, the word cancer. The CDI asked me once if I knew what it meant. I said that I knew there were different kinds. The CDI then proceeded to explain to me what cancer was. When I looked back at the hearing interpreter, s/he just kind of nodded and verified that the CDI was right. When I asked the hearing interpreter why s/he didn't do that, they said "Well, I fingerspelled the word, so I thought you knew what that meant." I'm just the patient, though, here about a hip surgery. I'm not the expert. There were several times throughout the appointment where the CDI kept having to jump in and explain things to me. It seemed like the CDI knew more than the hearing interpreter. |
|  | Pictures with short info |  | 95% | If you develop information access for all it will benefit Deaf people and if something benefits deaf people, it will benefit all. It's true. Except for Deaf folks with limited ASL but infographics are a good way to communicate information for everybody. Think about more of a Universal Design approach. |
|  | One on One |  | 62% | I have a similar thought to his. I think having options is good because this community doesn't use just one mode of communication. For example, we have two communication forms. Americans, North Americans are more wordy with how they describe, express and understand things, whereas other cultures and countries are more gestural and expressed in terms of relationship. So, for example, the information we provide in this country, or this community is definitely wordy, so that excludes many other people in other cultures who have different ways they take in information. Providing options makes the message more accessible. For example, people of lower social economic status feel turned off or annoyed when businesses use social media, electronic technology or other distanced forms of communication to communicate with their customers. They would much rather a human being come sit with them, spend time with them and interact with them, taking the time to get to know them as a person and explain what's going on. It's like watching the news on TV - it's hard to connect with from a distance. The challenge is to come at a message from different perspectives - delivering quality information at the different levels (registers) that will be understood by the community. |
|  | Articles |  | 29% | I use YouTube. For tick-related information, I like being able to see it (gestures removing tick) ... I know there's a lot of information in written English but seeing it explained visually on YouTube definitely helps me understand the written material better |
|  | Church |  | 10% | Church is also another place I’ve been to where I have learned some things, I went a couple of times. I haven't been able to go since corona virus but anyways they have interpreters at the church. I liked it and it was interesting. |
|  | Email |  | 29% | You can email me if you get any information, go ahead and email me with information. |
|  | Official Government Website |  | 19% | I think there should be more access for specific websites. not just from newly graduated people regurgitating information. I would prefer to have professionals signing content for high quality government websites, like the CDC, WHO, ....and I prefer to find the content there, rather than some person recycling information. |
| **Communication Efforts** |  |  |  |  |
| Provide Information in ASL/Need more ASL videos | |  | 100% | Normally there are maps of trails on those boards when you go somewhere and on the bottom, there will be a box of information explaining details of the trails like how many miles it is. There should be a QR code or barcode there that would allow people to open something on their phone that has all of that information in ASL. That way Deaf people can know how many miles the trails are, what they should look out for, if there are deer ticks or whatever else may be important for them to know. For example, if they do get bit by a tick, instructions on what they should do- remove it, disinfect it, bandage it up and go see your doctor. I'm not sure exactly what but that way all of those details would be available to them. There could also be another link to more translated videos that explain things in more detail if people are interested from that original page. I don't know the specifics but let’s say people are traveling not just in the states but in other countries like Puerto Rico or Equator? No how do you spell it? Interviewer: E-c-u-a-d-o-r Interviewee: Right Ecuador- maybe those areas have specific differences like a higher number of ticks or diseases. All of that information could translated in ASL and be attached to an itinerary people look up that is available to them online. People can know if there is a high number of ticks to the place they are traveling, how to prevent tick bites, what to do before you go, vaccinations, what diseases are prevalent, what symptoms are common. Deaf people who typically travel or go camping can have access to all of that information if those resources are translated and available in ASL. Deaf people will be equipped with this information and be able to tell their friends and community when they decide to go hiking or out traveling and word will spread. They will share this information and warn them about ticks and what to do because they saw this video in ASL and there will be a ripple effect. Those small efforts here and there, having those videos available will have a big effect in the long run. |
| Be involved |  | In response to how to be engaged with the Deaf community | 38% | go to events hosted by and for the Deaf community... take an asl class. be an advocate. if you see someone spreading misinformation about Deaf people, correct them. Those are a few ways you can be welcomed into the Deaf community as an active participant |
| Need to raise more awareness about ticks | |  | 52% | Do you feel there are resources available in ASL about tick diseases for the Deaf community? Interviewee: There needs to be more. |
| Work with deaf schools |  |  | 38% | Really, it's all about the big picture and there are many variables. For example, there is no standard definition of deafness. There are many different levels of hearing loss as well as many identities. I personally work in a college admissions department. How do I determine if students qualify as deaf or hard of hearing? If someone went to a school for the deaf, does that mean they automatically qualify? But what if that person technically has less of a hearing loss that someone who was mainstreamed? That mainstreamed person might be viewed as "not deaf enough" because they didn't go to a deaf school or get services. But how is that fair? The mainstreamed student could have lived in a rural area where there were no interpreters and the local professionals knew nothing about deafness. Do I tell that student too bad, you don't qualify? Other colleges could have different definitions or criteria for determining if a student qualifies. Our college [name of college] follows Congress' definitions of Deaf and Hard of Hearing, but how did Congress come up with their definitions? I'm sure that nobody that identifies as Deaf of Hard of Hearing helped Congress develop these definitions. So, in the grand scheme of things, the education system needs to develop a standard because it currently does not have one. There's no way to really know if the education at schools for the deaf is the same at hearing schools because hearing schools are highly varied. There's a vast difference between public schools and private schools. Some parents have the luxury to spend $100,000 a year on their child's education while other parents only have the option of sending their child to public school and hoping for the best. Like I said, there are just so many factors involved in education. Now, to take a deaf child and all the extra factors that should be considered for their education, and throwing them into a public school setting, on top of the other factors I just mentioned, oh my goodness! How can we help with all of that? It seems literally impossible. So, I forget where I was going with all of that. |
| Community engagement (Etic) |  |  | 52% | I think continuing to work in the community is important. An actual job. Many depend on SSDI, so their opportunities to get out and become visible are limited. They go into [name of store] and shop and they're in and out, they're not interacting with people. If someone's working there, they're not having an ongoing relationship and building relationships with other people that would help them learn more about you. |
| Virtual Community Engagement (Etic) | |  | 43% | I think that it's important to make the vlogs short and fun, this would make it more engaging. Hosting various activities one way to achieve this. (Interviewer asks her to elaborate) Maybe hosting a challenge would be one way to do this. There is a popular challenge called "bird box", I'm not sure if you have heard of this or not? Anyway, with this challenge people would blindfold themselves and do things like try to put makeup on. There was also the ice bucket challenge that brought awareness to an important issue. This challenge was something that took off, so many people shared that video. Maybe we could do a push-up challenge to virtually engage individuals from various Deaf communities. |
| They need to come in and interact with us | | In response to how to disseminate information | 29% | I think continuing to work in the community is important. An actual job. Many depend on SSDI, so their opportunities to get out and become visible are limited. They go into [name of store] and shop and they're in and out, they're not interacting with people. If someone's working there, they're not having an ongoing relationship and building relationships with other people that would help them learn more about you. |
| Certificated deaf interpreters |  |  | 48% | This one time I had a CDI, the CDI had arrived first. When the hearing interpreter came in, s/he looked surprised. S/he was asking who the CDI was and why the CDI needed to be there. The hearing interpreter didn't seem to understand why another person needed to be there to help him/her do their job. I could see the tension between the two of them, so I spoke up. I said I had requested the CDI so I could get the message in natively-signed ASL, which the hearing interpreter wouldn't be able to do. The interpreter tried to insist that they could sign in ASL, to which I had to explain that no, s/he can't sign in ASL with a native proficiency because s/he isn't Deaf. I ended the conversation with him/her by breaking eye contact and looking to the CDI. They started interpreting for me. The CDI started translating things clearly. I didn't even look at the hearing interpreter. I solely watched the CDI. At the end of the appointment, the hearing interpreter claimed they had never heard of a CDI before. I informed him/her that they were sorely behind the times. I think she or someone was asking questions to the provider, I realized there needs to be more CDI's available because there was only one CDI for all of the Deaf patients in that building at highland hospital. One interpreter! That's it, the interpreter said there is not many interpreters available so they have to split their time between the other patients. For example, I have an interpreter with me and it hits 4 o’clock, the interpreter interrupts and says "I have to go I have another appointment I have to be at!" And leaves. I am left waiting and waiting until the nurse or doctor comes in to check on me, but the interpreter still hasn't returned. They clearly show frustration on their face from waiting for the interpreter to come back so they can move forward with my appointment. I told them they were wasting my time and to call someone to get the CDI there! They seemed to figure it out and the doctor was able to come in. I was so shocked and asked the interpreter again, you really are the ONLY one available to interpret for ALL of the Deaf patients? The interpreter stated that all of the doctors were to follow the interpreters schedule based on the CDIs availability. So that means the Deaf persons schedule doesn't matter? Just the interpreters and I have to be available for that? Does that seem fair to you? |
| **Analytical Code** |  |  |  |  |
| Health literacy (etic) | (+) Strong Health Literacy |  | x | Some of it is related to my values that's another sad part of the story. [clarifying] Values. Values. That's the sad part. My values conflict with many of [name of news] news'. They are very much for themselves. They take care of themselves they hate anyone other than themselves. They don't like black people, they don't like gay people, they don't like a number of other people. They're very anti- everyone. My feeling is: I value the world. I think everyone contributes in some way. That's the world that I live in, that I support. Positivity and growth, that's my value. [name of news] is anti everyone and you see that. That's how I - that's what makes me not trust them. They're very oppositional - |
|  | (-) Low Health Literacy |  | x | What about other people that have the same values as Fox. How can they know that [name of news] is giving out wrong information, how do they develop that skill? |
| Health belief model and theory of reasoned action (etic) | |  | x | Interviewee: Probably many ways - interacting or working with others with similar values, sharing information. |
| Communication network (etic) |  |  | x | ...I can't speak for the rest of the world but I think a simple beginning would be to start with Social Media. You would at least have a couple of eyes on it, and then hopefully those right people would use their networks and it could would spread from there. That's where you have a network of specific people as administrators of posts with the ability to spread the message. But I do believe it really has to be a targeted approach. You can't just sent it out into the realm of social media and hope for the best, you really have to target specific people and ask for them to disseminate the information. It can't just be a blind dump of information in hopes that the right audience sees it. |
| Negative attitude toward deaf community | not enough visibility |  | 71% | Why is it that many deaf people often don't ask for clarification? Interviewee: I ask people why and they say that they're embarrassed to because they say that they are not smart. I try to tell them not to feel that way, but many deaf people say they are not intelligent and it embarrasses them. They don't want other people to look down on them and think they are less than. I feel the same way at times. I hate that feeling of people looking down on me because I have to ask for someone to repeat what they said and then I still don't understand. I can't stand seeing Deaf people just sitting there, smiling and not asking (the interpreter) for clarification when I know they don't understand. It eats me up inside. So, I might tell the interpreter like "hey, that person doesn't understand what you just said." Or, there are times when the interpreter has to add extra information for the interpretation to make sense in English, but they just talk and talk, and I have no idea what they're saying. So, I have to ask them to please sign as well as speak so I know that I'm being represented accurately. There are times I sign in ASL to the interpreter but then catch them making many mistakes when I read their lips while they interpret into English. I will jump in and say "that's not what I meant" and the interpreter replies back with "oh, I though that's what you said." Most of the interpreters in [name of city] are great, but some of them are not that good. |
| Positive attitude toward the deaf community | |  | 48% | I am the type of person to see the positive side of things. People here recognize us and our contributions to society and are even willing to learn sign language. I think that this has improved over the years. I know this positive attitude has to do with living in [name of city] and that not all cities and states experience this. In other areas Deaf people are still looked down upon and are viewed as incapable. What was the question again? (Interviewer clarifies question) There is more knowledge and awareness now. Deaf people are seen online and on TV. There are Deaf actors on TV shows now and this I think has helped change the attitude of people that are not Deaf. There is a larger presence of Deaf people in the media and I think this is a key component. There is also more visibility of interpreters in the mainstream media, such as press conferences. It brings awareness to the fact that Deaf people are present. If there is not an interpreter shown at a press conference, this thought may not come to mind to those watching. The fact that there is an interpreter shown forces people think about the Deaf audience and that Deaf people are capable of processing and understanding the same information and that we certainly are not incompetent. |
| Learning Ability (etic) | Autonomy (etic) | The individual have the ability to be independent and become knowledgeable about something when needed. | x | I was thinking about the reasons I am more assertive. It could be because of my education level and my place of employment- I work at a residential Deaf school. All of the employees talk about health care because we have that benefit provided to us through our job. We are more knowledgeable and understand the concerns. We engage in discussion with each other about these things and how the economy is impacting health care and what the sequalae of impacts are on ourselves. For others who are unemployed or have a job that doesn't provide them with health care it's very possible they aren't having these conversations and do not have the ability to discuss these matters with each other and potentially are not able to be assertive. I am sure there are some people who are curious and look things up on their own but many of them don't do that, so I think more access needs to be provided to those people. |
|  | No autonomy (etic) | The individual might do not care, too lazy, or do not know how to gain more knowledge. | x | When I think about my Deaf friends, they're all not very smart. I'm really concerned about when they go to medical appointments or the hospital, are they really understanding the interpreters? I'm not so sure. Back in the day, I used to offer to go with friends to help make sure they understood everything, but these days, having CDIs there to help is good enough. I realize that our Deaf community is diverse in their levels of understanding and their willingness to ask for clarification. Some will be passive and not ask because they don't want to be looked down on as less intelligent and have their pride hurt. They would rather nod and smile instead of fully understanding. Interviewer: I agree. That happens a lot in our community. |
